# Magnitude and variability of individual elbow load in repetitive baseball pitching

**DOI:** 10.1038/s41598-023-44333-x

**Published:** 2023-10-11

**Authors:** Bart van Trigt, Thomas van Hogerwou, Ton A. J. R. Leenen, Marco J. M. Hoozemans, Frans C. T van der Helm, DirkJan H. E. J. Veeger

**Affiliations:** 1 Department of Biomechanical Engineering, Delft University of Technology, Mekelweg 2, 2628 CD Delft, The Netherlands; 2 Department of Human Movement Sciences, Faculty of Behavioural and Movement Sciences, Vrije Universiteit Amsterdam, Amsterdam Movement Sciences, Van der Boechorststraat 9, 1081 BT Amsterdam, the Netherlands

**Keywords:** Mechanical engineering, Ligaments

## Abstract

In baseball pitchers the elbow is exposed to high and repetitive loads (i.e. external valgus torque), caused by pitching a high number of balls in a practice session or game. This can result in overuse injuries like the ulnar collateral ligament (UCL) injury. To understand injury mechanisms, the effect of repetitive pitching on the elbow load magnitude and variability was investigated. In addition, we explored whether repetitive pitching affects elbow muscle activation during pitching. Fifteen pitchers threw each 60 to 110 balls. The external valgus torque and electromyography of three elbow muscles were quantified during each pitch. Linear mixed model analyses were performed to investigate the effect of repetitive pitching. On a group level, the linear mixed models showed no significant associations of repetitive pitching with valgus torque magnitude and variability and elbow muscle activity. Significant differences exist between pitchers in their individual trajectories in elbow valgus torque and muscle activity with repetitive pitching. This shows the importance of individuality in relation to repetitive pitching. In order to achieve effective elbow injury prevention in baseball pitching, individual characteristics of changes in elbow load and muscle activity in relation to the development of UCL injuries should be investigated.

## Introduction

High performance in physical sports is closely related to musculoskeletal injuries. In baseball pitching, for instance, an important performance outcome is ball speed. For high performance, i.e. a high ball speed, a fast full-body motion is required, which exposes the musculoskeletal system to high mechanical load^1^. More specifically, in baseball pitching, the elbow is exposed to significant loads that might result in (overuse) elbow injuries. Most injuries at the elbow are on the medial side^2^, and more specifically on the ulnar collateral ligament (UCL). UCL injury rates in baseball pitching at all levels of play have gradually increased over the years^3,4^, as has surgery as a treatment option that involves reconstructing the UCL^5^, also known as the Tommy John surgery. To prevent injuries and the need for surgery among pitchers, it is crucial to understand the mechanisms of injury. This understanding can aid in the development of effective preventive measures.

Biomechanics could help to understand the injury mechanisms. It is stated that overuse injuries result from repetitive loading and cumulative bouts of activity and its interaction, defined as the mechanical fatigue phenomenon^6^. In terms of UCL injuries in baseball pitching, it is thus important to quantify the cumulative activity and the exposure to UCL load, preferably in terms of frequency, magnitude, and duration. Pitch count is an easy way to quantify the frequency and studies have shown that it is related to UCL injuries^7,8^. However, mechanical fatigue tests have shown that the risk of overuse injuries increases substantially with loading magnitude rather than loading cycles (i.e. frequency)^6^. The external valgus torque is frequently used as a proxy for UCL loading, as it is known that the UCL at least partially resists this torque^9–11^. Hence, a good measurement for the magnitude in baseball pitching is the external valgus torque. Thus, in baseball pitching, the pitch count and valgus torque, and its interaction seem important in relation to UCL injuries.

The external valgus torque around the elbow is generated by a rotational inertia component: a resistance to angular accelerations, and a translational inertia component: a resistance to linear accelerations^12^. The magnitude of the valgus torque depends on the position of the arm as well as the accelerations and is thus influenced by adjustments in pitching technique. Biomechanical changes and thus alteration in the external valgus torque might be related to changes in pitching technique because of a high number of balls in a practice session or during a match (i.e. repetitive pitching).

Three studies investigated the effect of repetitive pitching on the external valgus torque magnitude for different levels of play^13–15^. Darke et al.^13^ reported that the external valgus torque did not significantly change after throwing 75 balls in youth baseball pitchers^13^. Escamilla et al.^14^, who compared the external valgus torque between the last and the first inning of a simulated game, with an average of nine fastballs within each inning, also found no significant difference at the group level among collegiate pitchers. Murray et al.^15^ compared the valgus torque during a single pitch of the first inning with one from the last inning in professional baseball pitchers^15^, and also did not find significant differences on a group level.

Recently, we showed that within-individual load magnitude and variability differ among pitchers and that especially this variability might be related to overuse injuries^16^. A higher within-individual load variability increases the risk of sustaining an injury as, while the average load remains equal, more extreme values, closer to or even over the acute overuse injury level are likely to occur. Therefore, it is preferential to include multiple pitches in the analysis of an individual to investigate the effect of repetitive pitching on the elbow valgus torque magnitude and variability.

While investigating the association between repetitive pitching and UCL injuries with the external elbow valgus torque as elbow load measure, it should be noticed that the UCL is not the only structure that resists the external valgus torque. The elbow muscles can directly, via the flexor-pronator muscle group (FPM), and indirectly, via the co-contraction of the biceps and triceps muscles in relation to the joint geometry, counteract the elbow valgus torque^9,10^. The valgus torque is thus distributed over these structures, where the muscles might shield the UCL from high loads. In a previous study, we reported FPM activity at maximal external shoulder rotation, the critical moment when the peak external valgus torque occurs^17^. In addition, the biceps and triceps muscles were shown to be active at this critical moment^17^. A change in the muscle activation at the moment of the peak external valgus torque, due to for example a late onset or reduced muscle activity, could increase the UCL load while the external valgus torque remains the same. A prediction model by Sonne & Keir^18^ demonstrated that significantly more FPM muscle fatigue existed when the time between adjacent pitches was shorter. (8 seconds compared to 20 seconds)^18^, but possible changes in muscle activation in relation to repetitive pitching have not yet been subject of study.

The aim of this study is to determine whether there is a change in within-individual load magnitude and variability as an effect of repetitive pitching due to musculoskeletal fatigue-related kinematic changes during pitching. Based on the current literature it is expected that elbow valgus torque magnitude will not change with repetitive pitching, whereas the relationship of variability with repetitive pitching is difficult to predict. In addition, we intend to determine if and how repetitive pitching affects the activation of the FPM, biceps, and triceps during pitching.

## Methods

### Participants

Data were collected from fifteen healthy male baseball pitchers. Mean age was 24.5 years (SD 7.5), body height 191 cm (SD 5), body mass 79.4 kg (SD 9.2), and average ball speed 67mph (SD 4). Of the fifteen tested pitchers, 11 were right-handed. Most participants were pitching at a recreational level, with two participants playing at the highest level and one pitcher at the second highest level in the Netherlands. Specific individual information can be found in Table 1. None of the participants had experienced any musculoskeletal injuries in the past six months nor had they received elbow surgery in the past. The study protocol followed the guidelines stated in the Declaration of Helsinki^19^ and was approved by the Ethics Committee of the Delft University of Technology (HREC). Participants were informed of the procedure before the start of the measurements. Informed consent was obtained before involvement in the study.

**Table 1:** Table shows characteristics of each participant.

Participant number	Age	Level	Pitching experience	Type of pitcher	Body length	Body weight	Average ball speed
1	22	4th recreational level	10	Reliever	1.89	73.4	66
2	19	Highest professional league	11	Starter	1.93	85.9	74
3	25	4th recreational level	5	Starter	1.96	88.9	65
4	29	3rd recreational level	20	Reliever	1.91	78.5	67
5	44	6th recreational level	32	Starter	1.99	102.5	62
6	24	4th recreational level	12	Starter	1.85	71.0	68
7	18	1st recreational level	5	Reliever	1.91	75.9	70
8	17	1st recreational level	4	Starter	1.83	62.7	66
9	23	Highest professional league	5	Reliever	1.97	85.0	75
10	21	1st recreational level	12	Reliever	1.89	78.1	61
11	37	2nd recreational level	26	Starter	1.85	82.2	66
12	26	2nd recreational level	12	Starter	1.94	74.8	63
13	17	2nd professional league	5	Starter	1.92	81.9	73
14	24	4th recreational level	6	Starter	1.88	75.5	62
15	20	2nd recreational level	7	Starter	1.99	74.3	69

Age is in years. The level in the Netherlands range from low to high in the order of 6th recreational level, 4th recreational level, 3rd recreational level, 2nd recreational level, 1st recreational level, 2nd professional baseball league, highest professional baseball league. Pitching experience is in years. Body length is in meters and body weight is in kilograms. The average ball speed is in mph.

### Procedure

The measurements were performed at the indoor human movement laboratory of the Department of Human Movement Sciences at the Vrije Universiteit Amsterdam, The Netherlands. Fourteen reflective markers were placed on anatomical bony landmarks of the participants with double-sided tape (see Table 1). Electromyography (EMG) electrodes were placed on the skin of the throwing arm and an accelerometer was attached to the sternum below the incisura jugularis. The participants wore their own shoes, athletic shorts, and baseball glove, but no shirt. Prior to performing fastball pitches, participants had to perform maximum voluntary contractions for each muscle separately (MVC, see Table 1, Supplementary Materials). Participants gradually built-up muscle force and held this for 3 seconds. Each MVC was repeated three times. After performing their regular warm-up, the participants were instructed to pitch fastballs at full effort. Ten fastball pitches were performed within a block of pitches, with two minutes rest between each block. Before the start and between 10 blocks of pitches, the participants were asked about their self-perceived fatigue with the following text: “Place a vertical line on the visual analog scale shown below in which way you are overall fatigued”. The visual analog scale (VAS) ranged from totally not fatigued (0%) to extremely fatigued as possible (100%). Participants were instructed to stop when having thrown 110 fastballs or when their VAS score reached 80%. The minimum required number of pitches was 60. To investigate the effect of fatigue on variability, all the pitches were measured and included in the analyses. Participants pitched from a pitching mound (height 0.55m) towards a strike zone (height 0.71m; width 0.43m), at 18.66 m.

### Data acquisition

#### Kinematics and ball speed

Marker positions were recorded using an OptiTrack motion capture system with twelve cameras sampling at 120 Hz (OptiTrack Flex 13, OptiTrack™, Corvalis, United States). The OptiTrack system was calibrated to define camera position and orientation and to construct a convenient global coordinate system. The ball speed was measured behind the strike zone using a stalker pro radar gun (Stalker Radar, Plano, TX, USA).

#### Electromyography

Muscle activity of three elbow skeletal muscles of the throwing arm was measured using bipolar surface electromyography (sEMG). The flexor pronator mass (FPM), biceps brachii (BIC), and triceps brachii (TRI) muscles were measured (Table 2). The electrode locations were based on the SENIAM guidelines (Hermens et al. 1999)^20^. The reference electrode was placed on the clavicle of the non-throwing arm. Disposable bipolar electrodes (Ag-AgCl; 1 cm^2^ recording area; Blue Sensor Electrodes N-00-S, Ambu Inc., USA) were attached in the direction of the muscle fibers with 2 cm distance between the centers of the electrodes. Before the electrodes were attached, the skin was shaved and cleaned using alcohol. The electrode cables were fixated to the skin to avoid cable movement artifacts in the signal and to minimize the risk of loosening of the electrodes from the skin during pitch movement. The cables were connected to a BioPlux research device (Plux biosignals, Lisboa, Portugal), with 16-bits analog channels, a gain of 506, and an analog 25-500Hz band-pass filter. All consecutive fastball pitches of a participant were recorded in one EMG dataset at a sampling frequency of 2000Hz and locally stored on the BioPlux research device.

**Table 2:** Electromyography electrode position and orientation.

Muscle (group)	Electrode position and orientation	Electrode placement
m. biceps brachii (Bic)	On the line between the medial acromion and the fossa cubit at 1/3 proximal from the fossa cubit.	
Flexor pronator mass (FPM)	At 1/3 distal from the medial epicondyle. In the direction of the line between the medial epicondyle and the middle of the radial and ulna styloid	
m. triceps brachii (Tri) (lateral head)	At 1/2 on the line between the posterior crista of the acromion and the olecranon at 2 finger widths lateral to the line.	

### Data analysis

All data analyses were performed in Python (version 3.7, Python Software Foundation, https://www.python.org/).

#### Kinematics and inverse dynamics

The following bony landmarks on the throwing arm were used to construct an anatomical local coordinate system for the hand, forearm, and upper arm according to the ISB recommendations^21^: third proximal interphalangeal, ulna processes styloid, radius processes styloid, lateral humeral epicondyle, medial humeral epicondyle, and the acromion. Positions of the centers of mass and the moments of inertia were estimated according to Zatsiorsky^22^ and De Leva et al.^23^. The elbow joint angles were decomposed in the rotation order of ‘flexion/extension’—‘ab/adduction’ (floating angle)- ‘pronation-supination’ according to Grood and Sunday (1983)^24^. The shoulder angle was defined as the humerus in relation to the thorax. Maximal external shoulder rotation (MER) was obtained from the shoulder joint angles decomposed according to the y-x-y Euler decomposition (‘plane of elevation’-‘negative elevation’-‘axial rotation’)^25^.

The net joint forces and moments were calculated in the global coordinate system, using a top-down inverse dynamics analysis based on the Newton-Euler equation of motions. Subsequently, the elbow joint torque was expressed in the anatomical coordinate system of the elbow; positioned in the middle of the medial and lateral humeral epicondyles. The kinetics of the segments were calculated with the segment data and scaling factors of De Leva et al.^23^ and Zatsiorsky et al. (1990)^26^. A 2nd order polynomial function was fitted using five measured data points to obtain the exact magnitude of the peak value of the external valgus torque, which occurred around the moment of MER. The inverse dynamical model can be found here: https://github.com/ThomasBTHL/BTHL_public.

#### Electromyography

EMG signals were first separated into the ten-pitch series. Subsequently, these were cut into single pitches. The linear envelope was obtained by rectifying the EMG and applying a fourth-order bi-directional lowpass Butterworth filter of 20Hz. EMG data were normalized to the maximum values observed in the MVC data. To quantify the indirect effect of the biceps and triceps muscles, a co-contraction index (CCI) was calculated for the biceps and triceps muscle pair at each sample ($i$) according to Rudolph et al.^27^, see Eq. (1).

1 $${CCI}_{i}= \frac{{EMG}_{low,i}}{E{MG}_{high,i}}*\left({EMG}_{low,i}+E{MG}_{high,i}\right) .$$

An area under the curve (AUC) was calculated over a window of 150 ms for the normalized EMG data and the CCI. This window was chosen because the time between the events of foot contact to ball release is approximately 150 ms^28^, and includes the moment of the peak valgus torque. To represent the muscle activity as an indication of the timing of relative muscle force, the normalized EMG data was compensated with 50ms for the electromechanical delay (EMD)^29^. The moment from maximal external rotation to ball release is approximately 50 ms^17^, which is similar to the EMD. Therefore, the AUC window started at MER at 0ms and ranged back to – 150 ms to represent the timing of relative muscle force from foot contact to ball release.

#### Synchronization

The BioPlux device, containing the EMG signals and accelerometer data, did not contain the MER event. Therefore, it was synchronized with the OptiTrack system. The z-direction of the accelerometer, pointing forwards relative to the thorax, was synchronized with the forward acceleration of the trunk coordinate system. For each pitch, the data were synced on the peak linear accelerations and stored in a Python pickle.

#### Moving window approach

**Figure 1:**
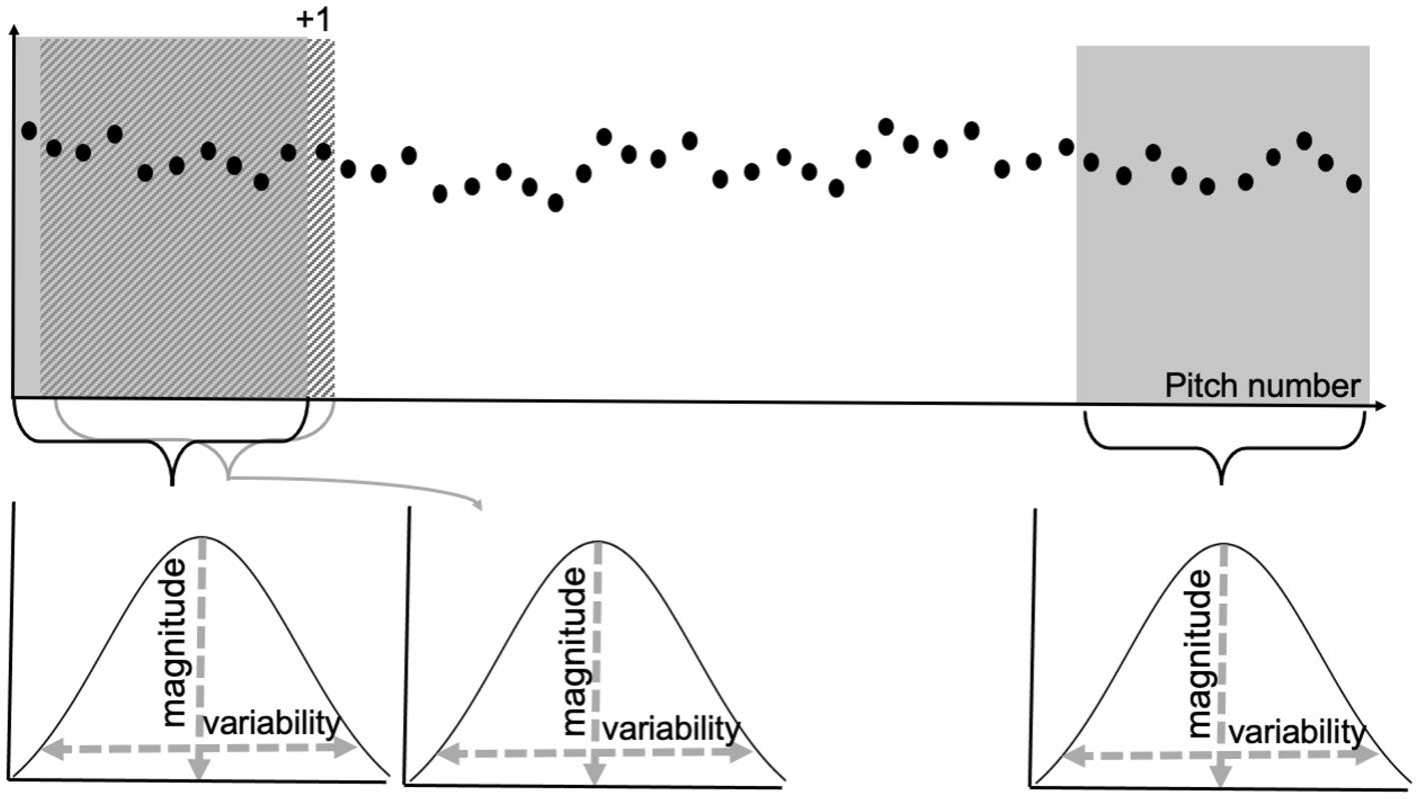
Visualization of the ten-pitch moving window approach moving over a single subsequent pitch.

In addition to ball speed, four outcome variables were analyzed in relation to repetitive pitching: the elbow valgus torque magnitude, valgus torque variability, the FPM AUC, and the biceps-triceps CCI. A moving window of ten pitches was applied to all variables and moved over a single subsequent pitch. The mean of the ten values within each window was used to quantify the trajectory of ball speed and the valgus torque magnitude, and the standard deviation of the ten values within each window was used to quantify the trajectory of the within-individual valgus torque variability over the individual sessions of 60-110 pitches (see Fig. 1). For the EMG outcome variables, the means of the ten values of the moving 10-pitch windows were quantified as the FPM activity and the biceps-triceps CCI.

### Statistical analysis

To statistically explore the relationship between ball speed and the four outcome variables and repetitive pitching five linear mixed models (LMM) were examined. The LMM deals with missing data and data of different samples^30^, which is an advantage as some participants indicated to be fatigued >80% after 60 balls, whereas others did not reach this level of fatigue after 110 balls. The fixed factor was the number of subsequent moving windows for each individual series of pitches and the random factor was the participant. Given the multilevel structure of the data (level 1 pitch window number nested in level 2 participants), it was considered necessary to build three models: (1) a basic model with a random intercept across participants, (2) a model with pitch window number as a predictor and random intercept across participants and (3) a model with pitch window number as a predictor, a random effect of pitch window number over participants (random slope) and random intercepts. To select the best-fitted model, the models were compared using a chi-square likelihood ratio test with a significant level of 0.05. If the models were significantly different, the model with the smallest AIC value was used. The maximum likelihood was used as the estimation method. The nlme package for R was used to perform the LMM analysis^31^. All statistical analyses were performed in R (version 4.2.0)^32^ and Rstudio (version 2022.2.0.443)^33^.

## Results

After visually inspection of the signals, for instance, due to missing markers, 951 pitches from 14 pitchers were included in the analysis. Participant 2 was removed because only thirteen pitches of the in total 60 pitches could be analyzed after preprocessing, which is not representative of repetitive pitching. Participant 1 did not have EMG data and was therefore not included in the EMG analyses.

### Ball speed

Ball speed was not significantly associated with window (or pitch) number *(*p = 0.76), indicating that ball speed remained constant throughout the pitching sessions of 60–110 throws. The visualization of these results can be found in Supplementary Materials.

### Magnitude and variability in relation to repetitive pitching

**Figure 2:**
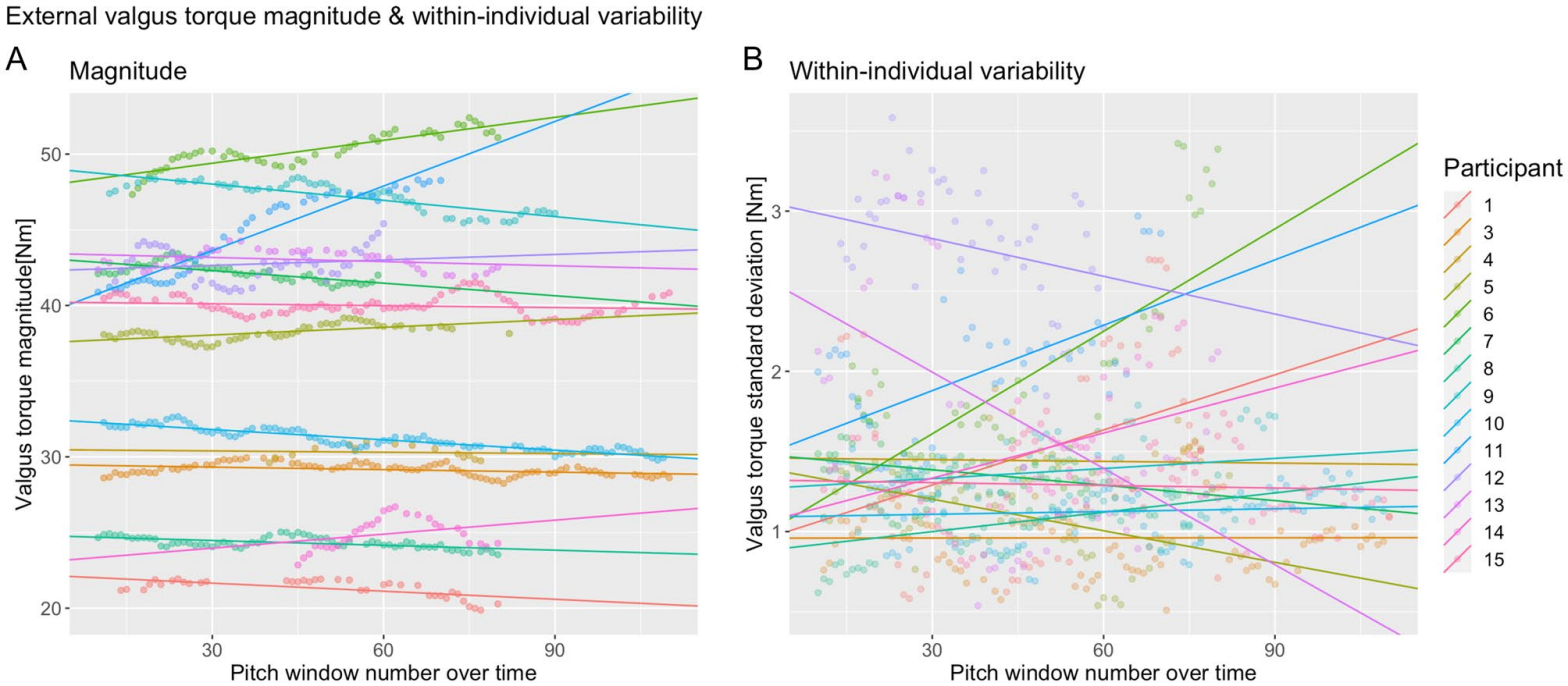
Panel (**A**) shows the relationship with the magnitude of the external valgus torque in relation to pitch number. Panel (**B**) shows the variability in relation to pitch number. Each colored line represents the modeled LMM intercept and slope of a participant.

The external valgus torque magnitude did not significantly change the fixed effect of pitch window number, and neither did the variability (Table 3). For both variables, the likelihood test showed that the linear mixed model with a random intercept and random slope was significantly the best model (Table 3, Supplementary Materials). Figure 2A shows the results of the model with across participants the significant random intercept (SD 8.71; 95% CI 6.01 12.61) and the significant random slope (SD 0.044; 95% CI 0.030, 0.064) for the external valgus torque magnitude. The linear mixed model of external valgus torque variability shows significant variance across participants for the random intercept (SD = 0.64; 95% CI 0.43, 0.94) and the random slopes (SD = 0.01; 95% CI 0.006, 0.015) (Fig. 2B). The standard deviation across participants for the slope was larger compared to pitch window number as a fixed effect, in both the magnitude and within-individual variability model. The likelihood test and the significant slope variances across participants indicate that the external valgus torque magnitude and variability depend on the individuals in relation to pitch window number.

**Table 3:** Table shows the results of the linear mixed model analysis of the predictor variable window number in association with the four outcome variables.

	ß	CI		t	Significance
Valgus torque					
Magnitude	0.0083	− 0.0148	0.0314	0.70	0.482
Variability	0.0017	− 0.0039	0.0073	0.59	0.553
EMG					
FPM (AUC)	− 1.9*10^-4^	− 5.0*10^-4^	1.1*10^-4^	− 1.22	0.223
CCI Triceps-Biceps	− 1.8*10^-4^	− 3.6* 10^-4^	6.6*10^-6^	− 1.89	0.059

ß is the slope of the linear relationship of the fixed effect. CI is the confidence interval with the lower and upper limits at respectively 2.5% and 97.5%. *p<0.05.

### Elbow muscle activity in relation to repetitive pitching

**Figure 3:**
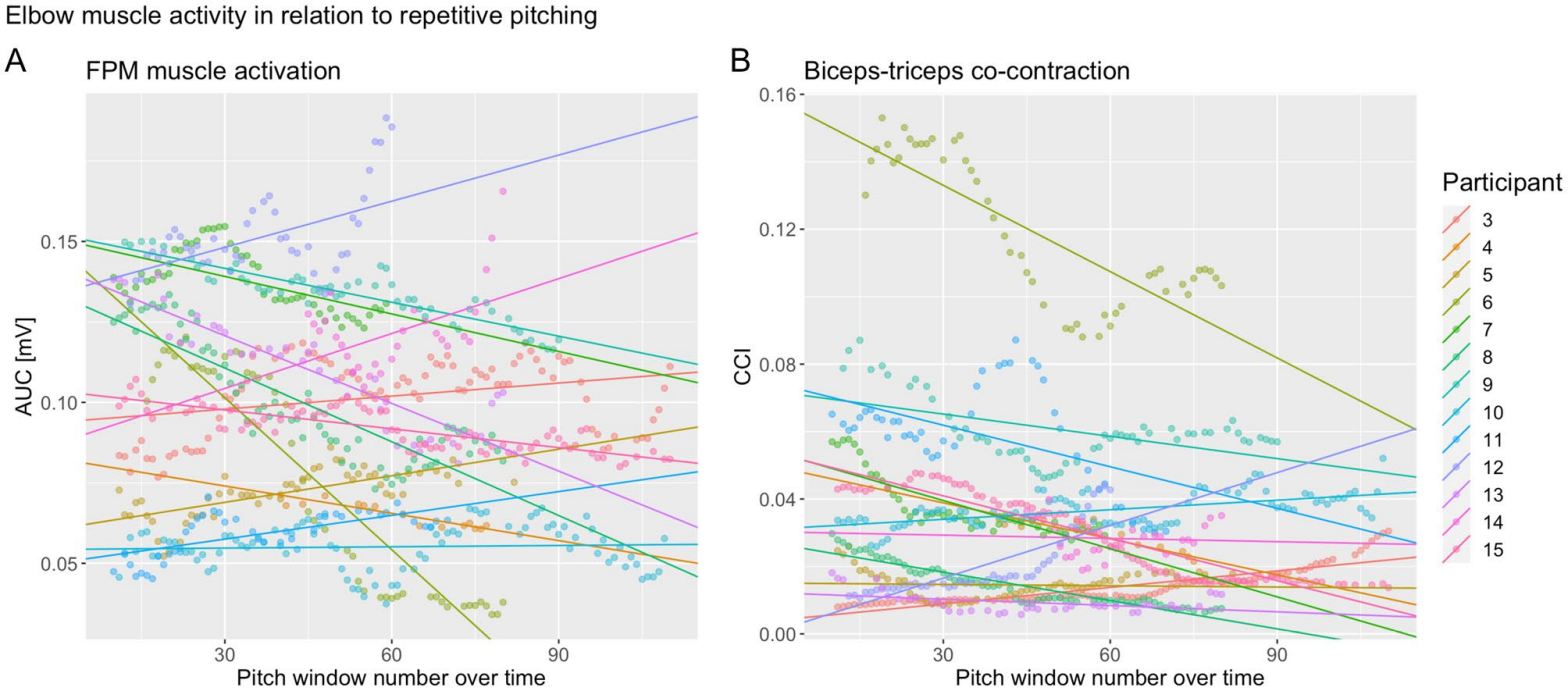
Panel (**A**) shows the relationship between the FPM activity area under the curve (AUC) with pitch window number. Panel (**B**) shows the biceps-triceps CCI in relation to the pitch window number. Each colored line represents the modeled LMM intercept and slope of a participant.

The FPM, biceps brachii and triceps brachii muscles showed activity in all participants during pitching. The FPM AUC activity was not significantly associated with the fixed effect of pitch number (Table 3). The underlying best-fitted model was the model with a random intercept and random slope across participants. Figure 3A shows the significant variance in intercepts across participants (SD = 0.06; 95% CI 0.04, 0.09), and the significant random slopes (SD = 0.0008; 95% CI 0.0005, 0.0012).

The biceps-triceps CCI showed a negative trend with pitch window number but was not significant (Table 3). Again, the best-fitted model was the model including random intercept and random slope. The intercept (SD = 0.04; 95% CI 0.027, 0.059) and slope (SD = 0.0003; 95% CI 0.0002, 0.0005) varied significantly across the participants (Fig. 3B).

These results indicate that on a group level repetitive pitching is not associated with FPM AUC activity and biceps-triceps CCI. The significant random slope difference across participants indicates that pitchers show a different FPM muscle activity and biceps-triceps CCI between each other in relation to pitch window number.

## Discussion

The aim of this study was to investigate if repetitive pitching influences the peak external valgus torque magnitude and variability during pitching, and to investigate the relationship between repetitive pitching and elbow muscle activation during pitching. The results showed no significant relationship between the external valgus torque magnitude and within-individual variability with repetitive pitching on a group level; but both variables showed significant variance in the association across participants. On a group level, the FPM activity was not significantly related to repetitive pitching. In addition, the biceps-triceps co-contraction index showed a trend but was not significant in relation to repetitive pitching. The FPM activity and the biceps-triceps co-contraction index showed significant variance in the association across participants. Thus, it is important to consider the individual differences for both the external valgus torque and elbow muscle activations in relation to repetitive pitching.

The external valgus torque in combination with pitch number is important in relation to UCL injuries, an higher external valgus torque with an increase in pitch number raises the chance of sustaining a UCL injury. Previous studies did not find an effect of repetitive pitching on the valgus torque by comparing the first and the last inning^13,14^. This is in line with our results, as no significant effect on group level was found between repetitive pitching and elbow valgus torque magnitude and variability, except that our results revealed that the individual association with repetitive pitching is very different across participants. The different responses across participants are an important finding as these can explain why no relationship with repetitive pitching was found on a group level. The importance of individuality has been shown earlier as the valgus torque magnitude and within-individual variability show considerable differences between pitchers in elbow load magnitude^34,35^ and in the load variability^16^. The results of the current study revealed also that individual differences are important in the association between elbow load and repetitive pitching.

The individual association of elbow load magnitude and within-individual variability with repetitive pitching emphasizes the importance of an individual approach in relation to the quantification of load and overload. This individual approach seems essential in relation to overuse injuries because it is hypothesized that pitchers who have a higher load magnitude and within-individual variability are at higher risk for sustaining an injury^36^. This, in combination with the mechanical fatigue phenomenon, where an increase in loading cycles (pitch count) and loading magnitude (valgus torque) increases the chance of damage, are important factors in overuse injuries. In terms of injury assessment, this knowledge is part of the larger complex puzzle to explain why one pitcher sustains an injury and another does not.

For injury prevention, the next step is to understand why one pitcher shows an increase and another a decrease or no changes in within-individual load magnitude and variability when performing relatively long sessions of repetitive pitching. Biomechanical variables earlier in the kinetic chain (such as leading leg knee extension and trailing leg knee flexion, and an earlier trunk rotation) are associated with an increased external valgus torque^37,38^. Alterations within an individual in these variables and other proximal intersegmental interactions could increase the external valgus torque magnitude and variability during repetitive pitching. In terms of injury prevention, it is thus important to investigate if these biomechanical variables can be trained to maintain a constant elbow load during repetitive pitching.

The FPM and the biceps-triceps CCI were active in all pitchers, with large inter-individual differences. It is difficult to explain the increase or decrease in muscle activation in pitchers. As a result of repetitive pitching, the AUC decrease in the subset of ten of our pitchers might reflect that they were not able to recruit the same amount of muscle fibers over the full duration of the experiment. On the other hand, pitchers who showed an increase might not have recruited all their muscle fibers in the beginning and compensate by an increase in muscle activation in association with repetitive pitching. The decrease could be especially dangerous, as it is known that pitchers with UCL insufficiency showed less activity in flexor carpi radialis and triceps muscles compared to uninjured pitchers^39^. Hence, several studies found a decrease in static grip force after repetitive pitching^40,41^, indicating an effect of repetitive pitching on the FPM strength. To conclude, the decrease in muscle activity and co-contraction index could be related to a reduction of produced muscle force. On the other hand, increased muscle activity could indicate the recruitment of additional muscle fibers, acting as a compensation mechanism. Electromyography is a noisy signal, and the results should thus be interpreted with caution, the next step is to investigate if the individual decrease in FPM activity and co-contraction levels are related to a decrease in muscle strength.

In baseball games, pitchers throw multiple pitch types such as breaking balls and fastballs. One limitation of this study is that the pitchers were instructed to throw fastballs only, because we were interested in the effect of repetitive pitching on the within-individual magnitude and variability of the elbow load and not in the differences between pitch types. Throwing a breaking ball produces less valgus torque^35^. Thus, the inclusion of other pitch types will show more variance in external valgus torque magnitude and variability. A lower valgus torque does not per definition imply a lower UCL load, because if the muscle force is decreased the UCL resists more stress. During breaking balls lower elbow muscle activations are reported^42^, assuming that the muscle force is also lower, which suggests that the shielding effect is different and might be even lower, in breaking balls compared to fastballs. A more in-depth comparison of muscular activity in different pitch types is therefore necessary.

The number of participants (n = 15) included in this study is comparable to other simulated game studies^14,43^. However, it is a relatively small sample size and therefore a limitation of this study. This is primarily due to the difficulty in recruiting pitchers to perform a fatiguing study, as such a number of pitches disrupt their training regime. In addition, in this study, the aim was to investigate the effect of repetitive pitching on the within-individual elbow load. Therefore, in comparison with other studies, we focused on analyzing many throws of individual pitchers, resulting in an enriched within-individual dataset. Another limitation is that the training load of the pitchers was not reported. This might have influenced the results as the study population was heterogeneous including recreational pitchers and professional pitchers. Overall, professional pitchers are exposed more frequently to higher loads and have better facilities and training schedules compared to recreational pitchers. This might have influenced the trajectory of muscle recruitment in relation to repetitive pitching. Future studies should investigate if training status and level of play influence the effect of repetitive pitching on muscle recruitment.

In this study, we investigated the association between repetitive pitching and elbow load during a single session. An injury can occur during only one single pitch when the peak load is higher than the UCL load capacity. However, most of the time it is a result of repetitive motion as the overall injury rate in baseball is 3.6 per 1000 athletes-exposures, with the elbow as the most injured part^44^. To prevent pitchers from injuries, monitoring the within pitcher development of the elbow load magnitude and frequency during every athlete’s exposure over multiple seasons is important. Wearables, like inertial measurement units (IMUs) can be used by individuals in the field to predict the elbow load^45^. Pitch count can be used as a substitute for loading frequency, while external valgus torque can be utilized to quantify the elbow load magnitude. Many studies used the external valgus torque to quantify the medial elbow load and used this as a proxy for UCL load^43,46^. However, as seen in this study, to quantify the UCL load it is important to consider the protection mechanism of the elbow muscles, especially individually in relation to repetitive pitching. Quantifying muscular activity with electromyography in a daily training session is upcoming but not yet possible. To mitigate pitching injuries in the future, a warning system can be developed based on monitoring the elbow load magnitude and frequency, and muscle activity. Future research in the field of UCL injuries should quantify the within-individual elbow load magnitude and variability over time.

## Conclusion

Repetitive pitching shows significant differences among pitchers in the relationship with the elbow within-individual load magnitude and variability, FPM activity, and the biceps-triceps co-contraction. The variation among pitchers could explain why no significant relationship was found on a group level. The differences in muscle activation among pitchers in relation to repetitive pitching show that the shielding effect of elbow muscles should be included when quantifying the UCL load and cannot be considered a constant variable. In the field of UCL injury assessment and especially injury prevention, our results show that it is important to measure the within-individual UCL load magnitude and variability in relation to repetitive pitching because these metrics could be part of the puzzle of understanding why one sustains an injury and another not. Future studies should investigate why some pitchers showed an increase in elbow within-individual load magnitude and variability load and others a decrease in relation to repetitive pitching and subsequently how it is causally related to UCL injuries.

### Supplementary Information

Supplementary Information.

Supplementary Tables.

## Data Availability

The datasets used and/or analysed during the current study available from the corresponding author on reasonable request.
