# Supplementary figures and images for: Magnitude and variability of individual elbow load in repetitive baseball pitching
Source: Sci Rep. 2023 Oct 11;13:17250. doi: 10.1038/s41598-023-44333-x (PMC10567693; doi:10.1038/s41598-023-44333-x)

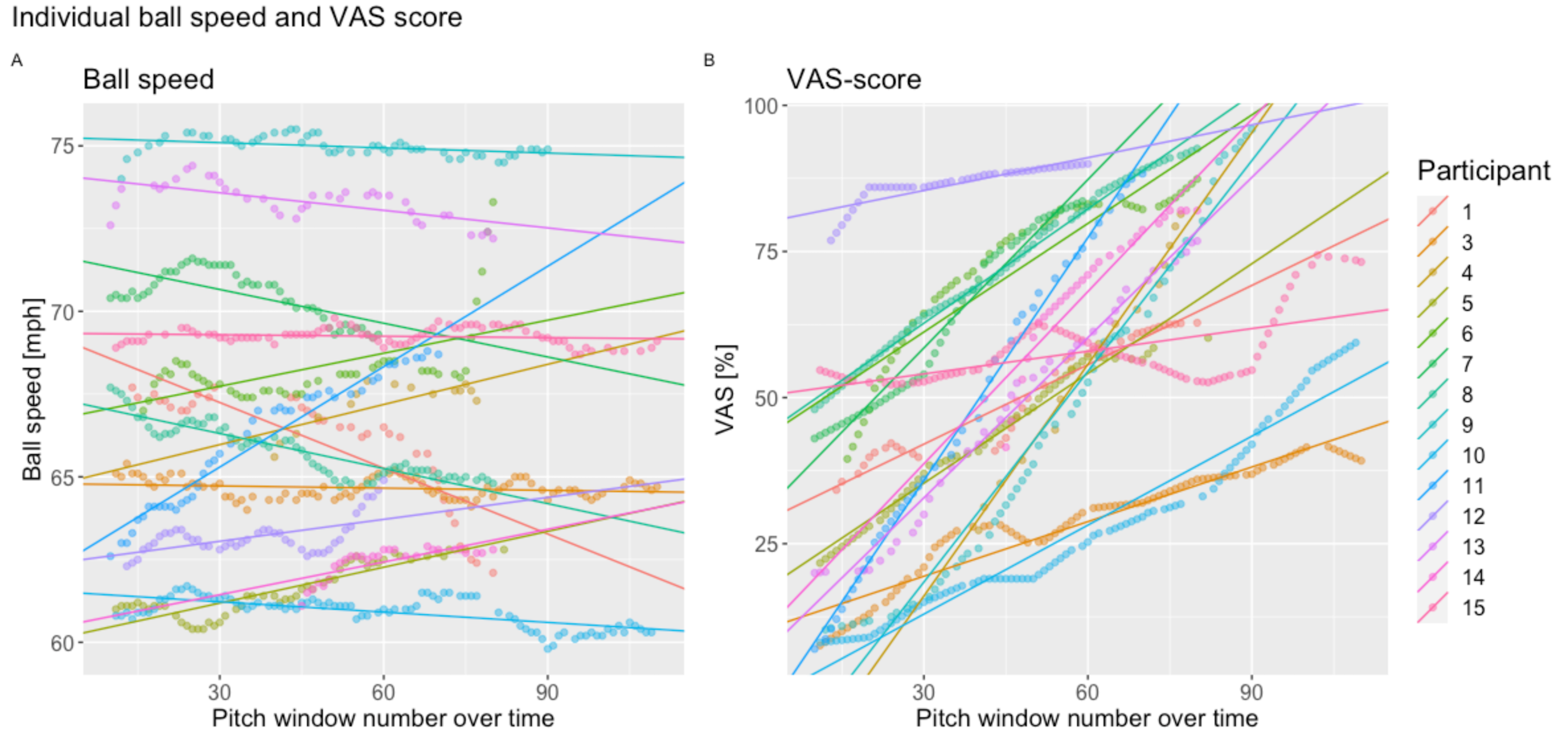

Supplement: Supplementary file 1 — Supplementary Information. [file 41598_2023_44333_MOESM1_ESM.png]
