# Supplementary material for: Magnitude and variability of individual elbow load in repetitive baseball pitching
Source: Sci Rep. 2023 Oct 11;13:17250. doi: 10.1038/s41598-023-44333-x (PMC10567693; doi:10.1038/s41598-023-44333-x)
Supplement: Supplementary file 2 — Supplementary Tables. [file 41598_2023_44333_MOESM2_ESM.docx]

# Supplementary Material

Table S.1: Maximal voluntary contact (MVC) tests. The gray arrow indicates the applied force direction of the participant. The black arrow indicates the direction of resistance

| **Muscle group** | **Maximal voluntary contraction test** | **Illustration** |
| --- | --- | --- |
| Flexor pronator group (FPM) | Seated or kneeling position in front of a table. With the forearm at approximately 90 °with respect to the upper arm. Participant performs a wrist flexion by pushing the hand palm against the bottom of a ground-fixed table. The table functions as static resistance. | 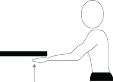 |
| M. biceps brachii | Seated or kneeling position in front of the table. With the forearm in approximately 90 °with respect to the upper arm, and the elbow rests on top of the table. One of the researchers applies a static resistance against the forearm while the participant performs an elbow flexion | 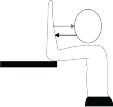 |
| M. triceps brachii | Seated or kneeling position in front of the table. With the forearm in approximately 90 °with respect to the upper arm, and the elbow rests on top  of the table. One of the researchers applies a static resistance against the forearm while the participant performs an elbow extension. | 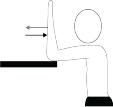 |

Table S.2. Loglikelihood results between the three different models for the four outcome variables. Model 1 was a basic model with a random intercept across participants. Model 2 included the pitch window number as predictor and random intercept across participants. Model 3 included pitch window number as predictor, a random effect of pitch window number over participants (random slope) and random intercepts.

|  | model 1 vs model 2 | model 2 vs model 3 |
| --- | --- | --- |
| Valgus torque magnitude | χ2(1) =2.47, p =0.115 | χ2(2) =703.7, p < .001 |
| Valgus torque variability | χ2(1) =3.79, p =0.054 | χ2(2) =139.8, p < .001 |
| FPM AUC | χ2(1) =63.84, p <0.001 | χ2(2) =614.7, p < .001 |
| Biceps-Triceps CCI | χ2(1) =64.8, p <0.001 | χ2(2) =521.8, p < .001 |
